# Supplementary material for: A novel infective endocarditis virulence factor related to multiple functions for bacterial survival in blood was discovered in Streptococcus sanguinis
Source: bioRxiv. 2024 Jul 3:2024.07.03.601854. Preprint. [Version 1] doi: 10.1101/2024.07.03.601854 (PMC11244957; doi:10.1101/2024.07.03.601854)
Supplement: 1 [file NIHPP2024.07.03.601854v1-supplement-1.pdf]

bioRxiv preprint doi: https://doi.org/10.1101/2024.07.03.601854; this version posted July 3, 2024. The copyright holder for this preprint (which was not certified by peer review) is the author/funder, who has granted bioRxiv a license to display the preprint in perpetuity. It is made available under aCC-BY 4.0 International license.

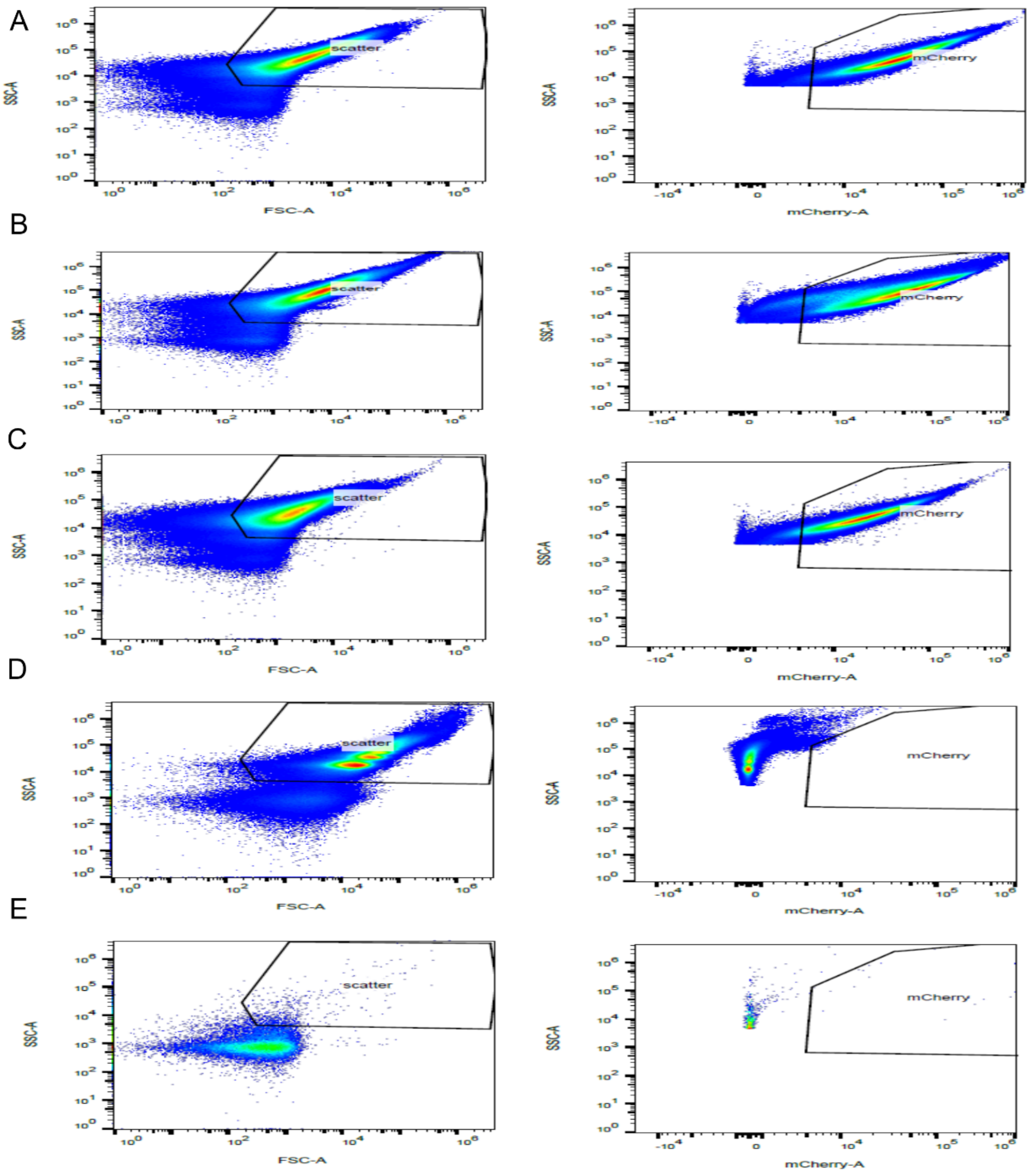

S1 Fig 1. Gate setting on Flow cytometry based on different controls. A: Red colored ΔSSA_0451 appeared in mCherry gate; B: Red colored SK36 appeared in mCherry gate; C: Red colored ΔSSA_0451C appeared in mCherry gate; D: Blood only control not appear in mCherry gates; E: Sterile water control also not appeared in mCherry gates.

bioRxiv preprint doi: https://doi.org/10.1101/2024.07.03.601854; this version posted July 3, 2024. The copyright holder for this preprint (which was not certified by peer review) is the author/funder, who has granted bioRxiv a license to display the preprint in perpetuity. It is made available under aCC-BY 4.0 International license.

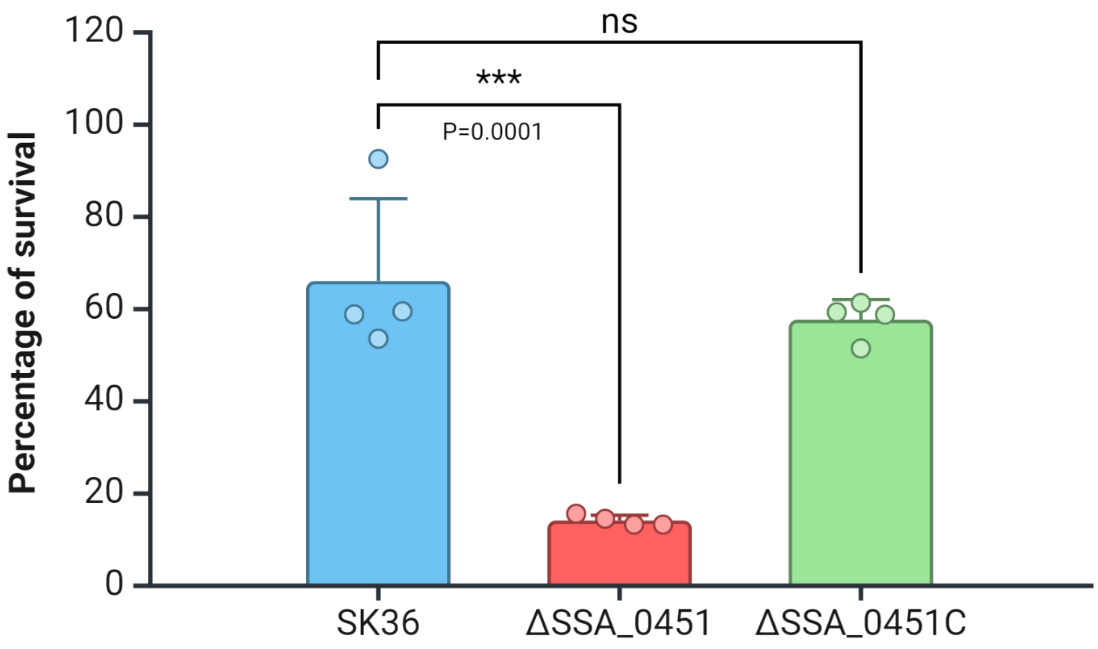

S1 Fig 2. Survival of ΔSSA_0451 and ΔSSA_0451C after 30 min was shown based on CFU count. Values are ± SD, n=4.

bioRxiv preprint doi: https://doi.org/10.1101/2024.07.03.601854; this version posted July 3, 2024. The copyright holder for this preprint (which was not certified by peer review) is the author/funder, who has granted bioRxiv a license to display the preprint in perpetuity. It is made available under aCC-BY 4.0 International license.

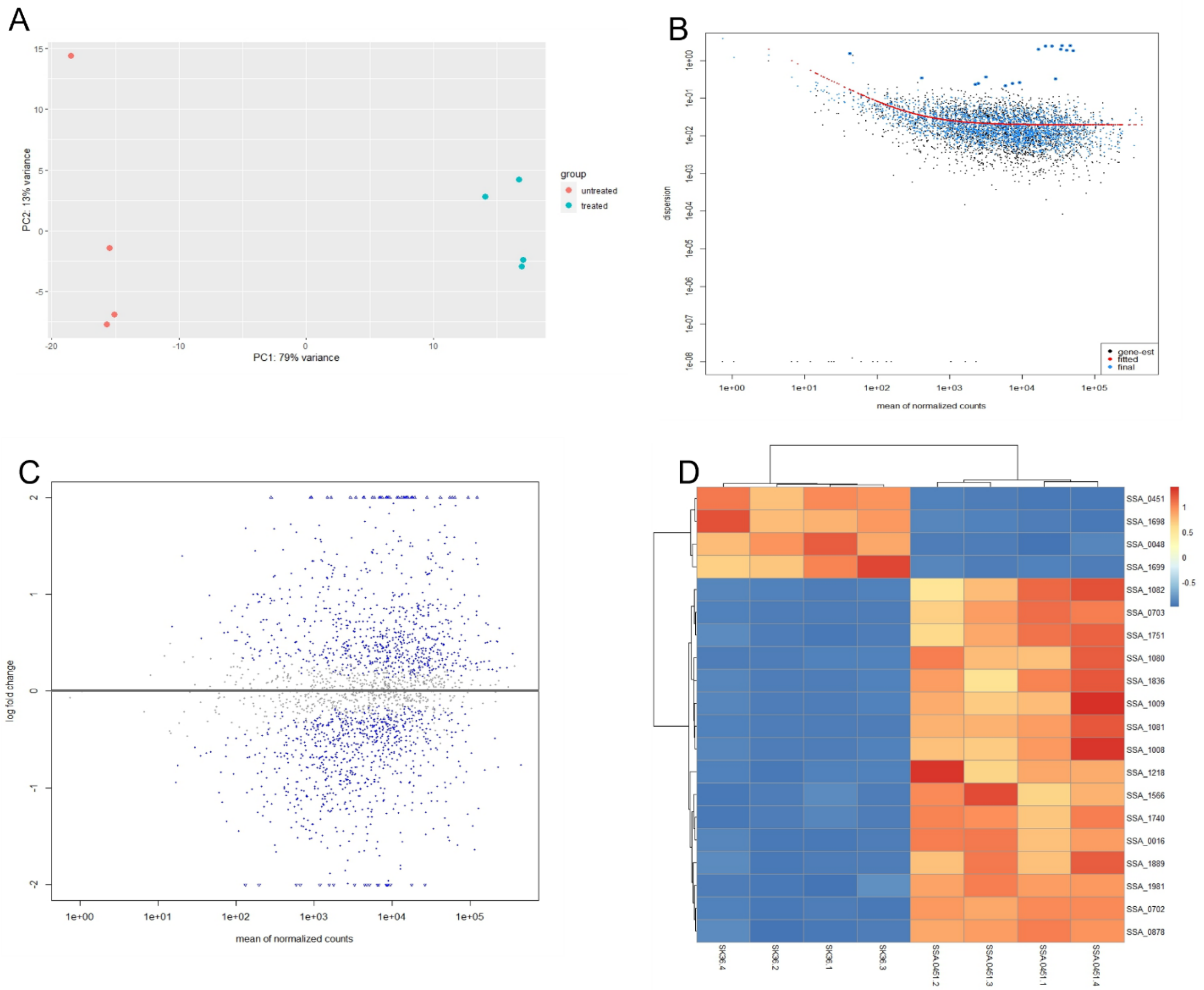

S1 Fig 3. RNA-Seq analysis of ΔSSA_0451 and SK36 showed upregulated and downregulated genes. A: PCA plot shows the distribution of SK36 and ΔSSA_0451 biological replicates. B: Dispersion analysis of SK36 and ΔSSA_0451 based on mean normalized counts., C: MA Plot shows the distribution of upregulated and downregulated genes based on logfold change and mean normalized counts. D: Top upregulated and downregulated genes based on Z-score.

bioRxiv preprint doi: https://doi.org/10.1101/2024.07.03.601854; this version posted July 3, 2024. The copyright holder for this preprint (which was not certified by peer review) is the author/funder, who has granted bioRxiv a license to display the preprint in perpetuity. It is made available under aCC-BY 4.0 International license.

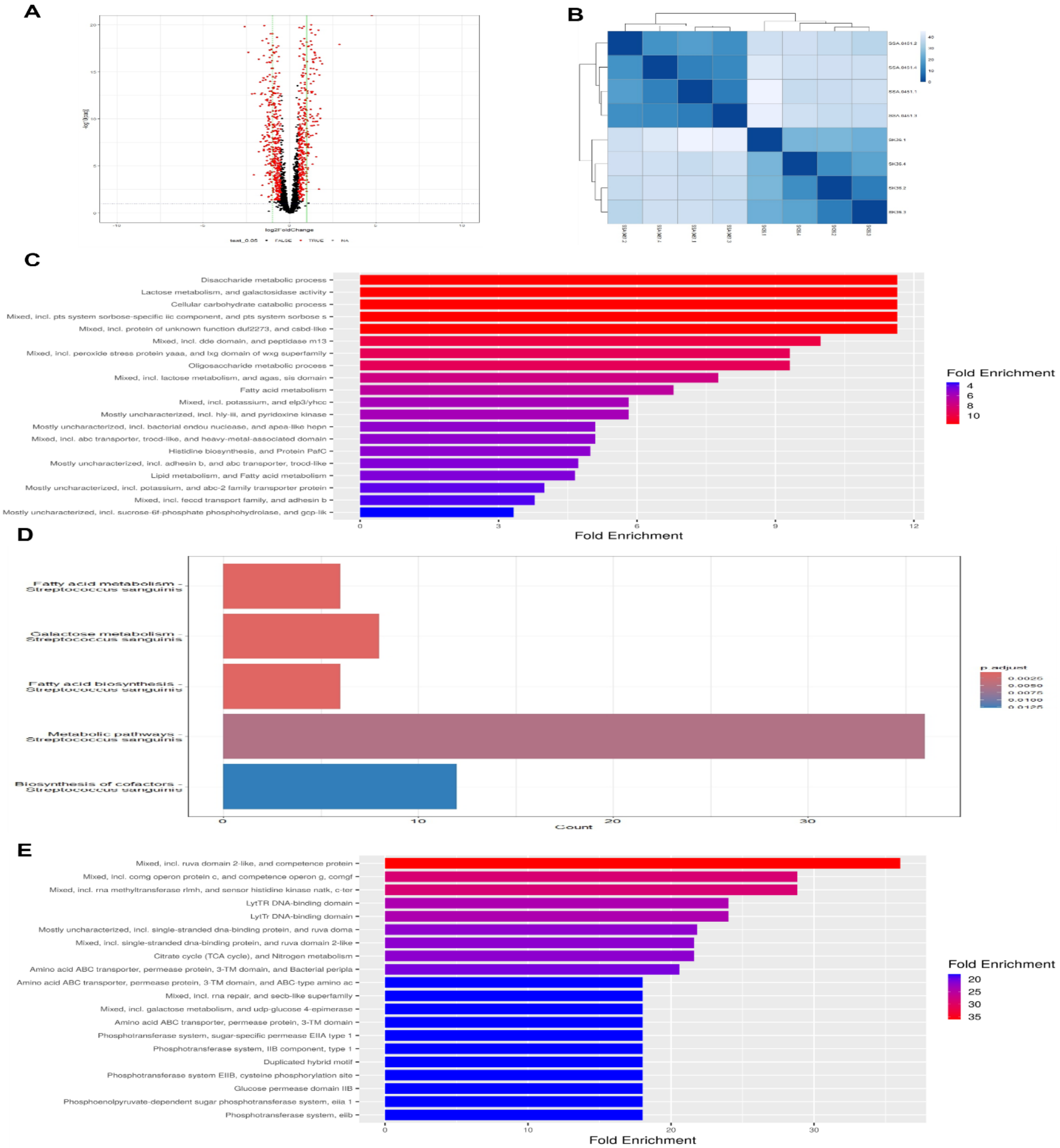

S1 Fig 4. RNA-Seq analysis of ΔSSA_0451 and SK36. A: Volcano plot shows upregulated and downregulated genes based on their log2 fold value. B: Heat Map shows the difference between SK36 and ΔSSA_0451 replicates. C: The Gene ontology analysis of -1.5 log2fold genes shows the downregulated biological process. D: KEGG pathway analysis of -1.5 log2fold decreased genes shows the downregulated pathways. E: The Gene ontology analysis of 1.5 log2fold increased genes shows the upregulated biological process.

bioRxiv preprint doi: https://doi.org/10.1101/2024.07.03.601854; this version posted July 3, 2024. The copyright holder for this preprint (which was not certified by peer review) is the author/funder, who has granted bioRxiv a license to display the preprint in perpetuity. It is made available under aCC-BY 4.0 International license.

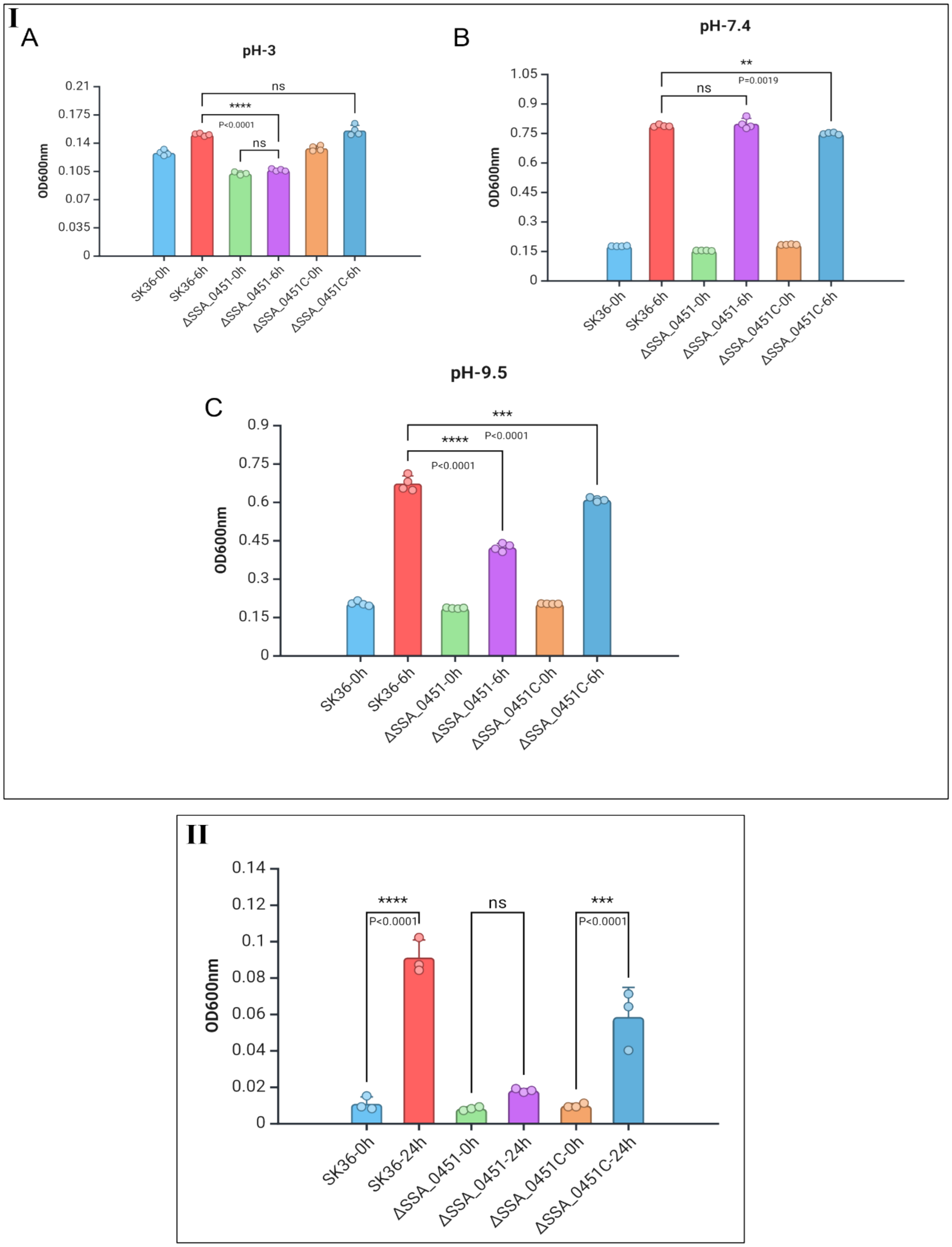

S1 Fig 5. The SSA_0451 gene is necessary for *S. sanguinis* to survive under stress. I-A-C: Survival of ΔSSA_0451 in different pH. II: Survival of ΔSSA_0451 in bile salt.

bioRxiv preprint doi: https://doi.org/10.1101/2024.07.03.601854; this version posted July 3, 2024. The copyright holder for this preprint (which was not certified by peer review) is the author/funder, who has granted bioRxiv a license to display the preprint in perpetuity. It is made available under aCC-BY 4.0 International license.

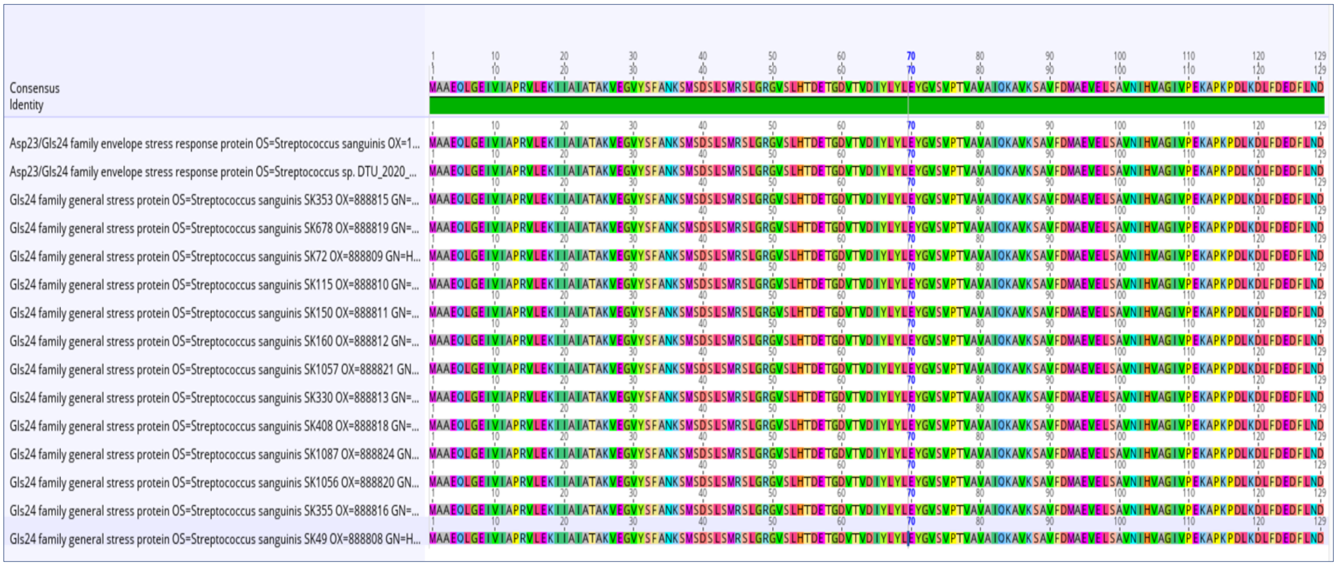

S1 Fig 6 Protein homologous search. The Protein homologous search displayed that this protein is homologous to the alkaline shock protein-23 (Asp-23) or Gls24 family.

bioRxiv preprint doi: https://doi.org/10.1101/2024.07.03.601854; this version posted July 3, 2024. The copyright holder for this preprint (which was not certified by peer review) is the author/funder, who has granted bioRxiv a license to display the preprint in perpetuity. It is made available under aCC-BY 4.0 International license.

S1 Table 1. PTS system's mannose-specific IIC component-related genes were downregulated in RNA-Seq analysis of ΔSSA_0451 and SK36.

Locus	log2 foldChange	pvalue	padj
**SSA_0219**	-3.20344	2.15E-83	2.10E-81
SSA_0220	-3.03231	8.50E-37	2.45E-35
SSA_0221	-2.86507	5.28E-36	1.43E-34
SSA_0222	-2.82302	5.85E-30	1.16E-28
SSA_0224	-2.92698	3.75E-22	4.87E-21
SSA_2023	-2.38917	8.11E-19	8.61E-18

The values were obtained from four biological replicates.

bioRxiv preprint doi: https://doi.org/10.1101/2024.07.03.601854; this version posted July 3, 2024. The copyright holder for this preprint (which was not certified by peer review) is the author/funder, who has granted bioRxiv a license to display the preprint in perpetuity. It is made available under aCC-BY 4.0 International license.

S1 Table 2. Strains and plasmids used in this study.

Strain and plasmids	Description	Reference
S. sanguinis SK36	Human plaque isolate	[39]
JFP36	Em^r; Δ0169 : : pSerm, derived from SK36	[49]
JFP56	Sc^r; Δ0169 : : aad9, derived from SK36	[49]
ΔSSA_0451	SK36, insert-deletion of SSA_0451 gene, Km^r	[50]
pVMTeal-Plasmid	Gram-positive replicative plasmid carrying *S. mutans* ldh promoter and codon-optimized gene for mTFP1; Em^r	[43]
pVMcherry-Plasmid	Gram-positive replicative plasmid carrying *S. mutans* ldh promoter and codon-optimized gene for mCherry; Em^r	[43]

Km, kanamycin; Em, erythromycin; Sc, spectinomycin.

bioRxiv preprint doi: https://doi.org/10.1101/2024.07.03.601854; this version posted July 3, 2024. The copyright holder for this preprint (which was not certified by peer review) is the author/funder, who has granted bioRxiv a license to display the preprint in perpetuity. It is made available under aCC-BY 4.0 International license.

S1 Table 3. Primers used in this study.

Primer name	Primer Sequence
**ΔSSA_0451 creation: SSA_0451 Knockout with Kanamycin resistance (Km)**	
SSA_0451-F1-Km	TTTGGCTGCTACTACAATCACTATG
SSA_0451-R1-Km	GCCATTTATTCCTCCTAGTTAGTCATGCCATAGTTATTACCTCTTCTAT
SSA_0451-F2-Km	TGACTAACTAGGAGGAATAAATGGCTAAAATGAGAATAT
SSA_0451-R2-Km	CATTATTCCCTCCAGGTACTAAAACAATTCATCCAGT
SSA_0451-F3-Km	GTTTTAGTACCTGGAGGGAATAATGGCACCGAAGCCAGACTTGAAAG
SSA_0451-R3-Km	TAACTGGTTCTTGCTTGTCTTTTTC
**ΔSSA_0451C creation: SSA_0451 Complementation with Spectinomycin resistance (SC)**	
SSA_0451C-F1-Sc	TTTGGCTGCTACTACAATCACTATG
SSA_0451C-R1-Sc	AGATTAAAAAAATTATAAGGCCGCCGCCGTCATTGAGGAAGTCCTC
SSA_0451C-F2-Sc	CCGCTCTAGAACTAGTGGATCC
SSA_0451C-R2-Sc	CAATTTTTTTATAATTTTTTTAATCTG
SSA_0451C-F3-Sc	CCACTAGTTCTAGAGCGGTATACTGTTGGAATCCAGAAGAGG
SSA_0451C-R3-Sc	TAACTGGTTCTTGCTTGTCTTTTTC
**SSA_0451 Complementation with Erythromycin resistance (Em)**	
SSA_0451C-F1-Em	TTTGGCTGCTACTACAATCACTATG
SSA_0451C-R1-Em	TCATGTAATCCTCCTAGTTAGTCATTATTCAGTCATTGAGGAAGTCCTC
SSA_0451C-F2-Em	TGACTAACTAGGAGGATTACATGAACAAAAATATAAAATATTCT
SSA_0451C-R2-Em	CATTATTCCCTCCTAGAATTATTTCCTCCCGTTAAATAATAG
SSA_0451C-F3-Em	AAATAATTCTAGGAGGGAATAATGTATACTGTTGGAATCCAGAAGAGG
SSA_0451C-R3-Em	TAACTGGTTCTTGCTTGTCTTTTTC

Km, kanamycin; Em, erythromycin; Sc, spectinomycin.

bioRxiv preprint doi: https://doi.org/10.1101/2024.07.03.601854; this version posted July 3, 2024. The copyright holder for this preprint (which was not certified by peer review) is the author/funder, who has granted bioRxiv a license to display the preprint in perpetuity. It is made available under aCC-BY 4.0 International license.

S1 Table 4. Primers used for qPCR study

Primer name	Primer Sequence
QPCR-SSA_0260F	AAACTGGCGGTAATGCTTGG
QPCR-SSA_0260R	AAATGACGTCAACGCCATCG
QPCR-SSA_0261F	TTATCATTCTGCGCGGCATG
QPCR-SSA_0261R	AGGCCAAAAGTCCGAAAGTG
QPCR-SSA_0262F	TCTTGATTGCTCGCTGCTTG
QPCR-SSA_0262R	TCTTCGCTGACAGAGTCAATCC
QPCR-SSA_0259F	TGCTGTCATGCTCTCTGACTAC
QPCR-SSA_0259R	GCCATTCATTGATGAGGACAGC
QPCR-SSA_2186F	CGGCGCAATTCCAACATTTC
QPCR-SSA_2186R	TTTGCCATGTCAGCAGCAAG
QPCR-SSA_1533F	TGGTCGTAAGCCAAACGTTC
QPCR-SSA_1533R	ACAGCAATGAAGCCACGTTC
QPCR-SSA_1745F	AAGGCGCTGTTAAAGAAGGC
QPCR-SSA_1745R	TCTACAGCACCTTCTACAGCTC
QPCR-SSA_2148F	TTCAGGTTTGCTGGCTGTTG
QPCR-SSA_2148R	ATTCCGCAACCACATTCAGG
QPCR-SSA_0721F	TGCATCATGACAAGCACCAC
QPCR-SSA_0721R	TTGTTGATGACTGCCTGACG
QPCR-SSA_1523F	ACGACCTTTCCTCGATTTGC
QPCR-SSA_1523R	TCGGCTGACCAGAAACTTTG
QPCR-SSA_2052F	TTGACTTTTGGGCCACTTGG
QPCR-SSA_2052R	TTCCTCTGAGAGCTTGTCAAGG
QPCR-SSA_1220F	AAATGGCCGTGAGCGAATTG
QPCR-SSA_1220R	ATGCGTTTTTCCTGCACCAG
QPCR-SSA_1080F	AAACTGCGACGAGTTCATGG
QPCR-SSA_1080R	TCAAGCTTGCTGCCATCTTG
QPCR-SSA_0716F	GTTTGCCTTGTCTGGTATGCAG
QPCR-SSA_0716R	TGAAAAAGGCAGCTGCAAGG
QPCR-SSA_1698F	TGGTGTTCGTTCAGCTTTGG
QPCR-SSA_1698R	TCCGCATGGATGAAAGCTTC
QPCR-SSA_1699F	ATGGGAGCTGAAATCGTTGG
QPCR-SSA_1699R	TTGTTGAGCATGTCGACTCG
QPCR-SSA_1938F	ACGCGTGATTTCGAAAAGGC
QPCR-SSA_1938R	AACAACCGCATTGGCCAAAG
QPCR-SSA_1941F	AATGGGCGGCTTTTGTATCG
QPCR-SSA_1941R	AGGCCCTTTTGCATCACTTG
